# Distinct Functional Connectivity Signatures of Impaired Social Cognition in Multiple Sclerosis

**DOI:** 10.3389/fneur.2020.00507

**Published:** 2020-06-25

**Authors:** Sabrina Golde, Josephine Heine, Jana Pöttgen, Maron Mantwill, Stephanie Lau, Katja Wingenfeld, Christian Otte, Iris-Katharina Penner, Andreas K. Engel, Christoph Heesen, Jan-Patrick Stellmann, Isabel Dziobek, Carsten Finke, Stefan M. Gold

**Affiliations:** ^1^Charité – Universitätsmedizin Berlin, Klinik für Psychiatrie und Psychotherapie, Berlin, Germany; ^2^Clinical Psychology and Psychotherapy, Department of Education and Psychology, Freie Universität, Berlin, Germany; ^3^Charité – Universitätsmedizin Berlin, Klinik für Neurologie, Berlin, Germany; ^4^Institute of Neuroimmunology and Multiple Sclerosis (INIMS), Unversity Medical Center Hamburg-Eppendorf (UKE), Hamburg, Germany; ^5^Department of Neurology, University Medical Center Hamburg-Eppendorf (UKE), Hamburg, Germany; ^6^Berlin School of Mind and Brain, Humboldt Universität zu Berlin, Berlin, Germany; ^7^Department of Neurology, Medical Faculty, Heinrich Heine University, Düsseldorf, Germany; ^8^Department of Neurophysiology and Pathophysiology, University Medical Center Hamburg-Eppendorf, Hamburg, Germany; ^9^APHM, Hôpital de la Timone, CEMEREM, Marseille, France; ^10^Aix Marseille Univ, CNRS, CRMBM, UMR 7339, Marseille, France; ^11^Institute of Psychology, Humboldt Universität zu Berlin, Berlin, Germany; ^12^Charité – Universitätsmedizin Berlin, Medizinische Klinik m.S. Psychosomatik, Berlin, Germany

**Keywords:** multiple sclerosis, social cognition, functional connectivity, emotion recognition, fusiform gyrus

## Abstract

**Objective:** Multiple sclerosis (MS) is characterized by impairments in basic cognitive functions such as information processing speed as well as in more complex, higher-order domains such as social cognition. However, as these deficits often co-occur, it has remained challenging to determine whether they have a specific pathological basis or are driven by shared biology.

**Methods:** To identify neural signatures of social cognition deficits in MS, data were analyzed from *n* = 29 patients with relapsing–remitting MS and *n* = 29 healthy controls matched for age, sex, and education. We used neuropsychological assessments of information processing speed, attention, learning, working memory, and relevant aspects of social cognition (theory of mind, emotion recognition (ER), empathy) and employed neuroimaging of CNS networks using resting-state functional connectivity.

**Results:** MS patients showed significant deficits in verbal learning and memory, as well as implicit ER. Performance in these domains was uncorrelated. Functional connectivity analysis identified a distinct network characterized by significant associations between poorer ER and lower connectivity of the fusiform gyrus (FFG) with the right lateral occipital cortex, which also showed lower connectivity in patients compared to controls. Moreover, while ER was correlated with MS symptoms such as fatigue and motor/sensory functioning on a behavioral level, FFG connectivity signatures of social cognition deficits showed no overlap with these symptoms.

**Conclusions:** Our analyses identify distinct functional connectivity signatures of social cognition deficits in MS, indicating that these alterations may occur independently from those in other neuropsychological functions.

## Introduction

Multiple sclerosis (MS) is an inflammatory, demyelinating, and neurodegenerative disease (1). In addition to the well-known impairments in visual and motor systems (2), it is characterized by impairments in learning, memory, information processing speed (3), mood disturbances (4), fatigue (5), and impairments in other domains such as social cognition. These symptoms often co-occur in individual patients (6). Thus, parsing the specific neuropathological pathways that underlie each of these symptoms has remained challenging, impeding the development of targeted treatment approaches (7). One suitable approach to address this problem is the search for underlying large-scale functional network signatures. This assumes that even if symptoms are correlated on the behavioral level, they should map onto distinct neural networks if they arise independently but would show overlapping signatures if they are driven by a shared pathobiology.

MS targets the interconnected cortical network rather than single regions (8). It is now becoming increasingly clear that social cognition, which relies on intricate coordination of large-scale brain networks (9), is strongly affected by MS and might be even more sensitive to MS-related damage than classical neuropsychological deficits (10). Recent meta-analyses report that MS deficits in this domain were of similar or even greater magnitude compared to those observed in classical areas of neurocognition (10, 11).

Social cognition refers to our capacity to understand and adequately respond to the mental states of others, therefore forming the basis for interpersonal relationships and social support. For MS patients, social cognitive skills are of critical importance with regard to their individual disease burden. The capacity to understand and adequately respond to the mental states of others forms the basis for interpersonal relationships and social support of patients. Moreover, a decline in these skills has been linked to deteriorating social and psychological quality of life (12).

Neuroimaging studies in MS have successfully linked neuropsychological deficits such as impaired processing speed or decreased memory function to subtle alterations in neural subnetworks (13). In contrast, the neural network changes associated with social cognition deficits in MS remain poorly understood. Early studies observed altered regional activation during social cognition tasks in brain areas relevant to affective processing (e.g., amygdala) as well as top-down control (e.g., ventrolateral prefrontal cortex) (14–16). More recently, structural MRI studies have linked social cognition deficits to lesion load distributed across the brain (17) and to atrophy of a variety of brain structures in the prefrontal, temporal, parietal, and occipital cortices (18, 19). Notably, amygdala lesions have repeatedly been found to be a major predictor of social cognitive deficits (19). However, given their complexity, social cognitive processes rely on intricate coordination of large-scale brain networks (9), rather than on narrowly defined brain areas. In line with this, social cognition deficits in MS were found to be correlated with subtle but widely distributed white matter damage as measured by diffusion tensor imaging (20). It remains unknown if MS-associated social cognitive deficits are simply an epiphenomenon or constitute a primary impairment with specific pathophysiological substrates. Neural origins, particularly regarding functional brain organization, need further deciphering. Resting-state functional connectivity MRI may be a particularly powerful method to study subtle, large-scale neural networks (21), as it is sensitive to complex pathology, neural re-organization, and their relation to behavioral status (22).

We therefore employed functional connectivity analysis to investigate neural network signatures associated with social cognitive performance in multiple sclerosis (MS). In particular, we examined the specificity of social cognitive deficits in relapsing–remitting MS. We pursued the following three consecutive aims: (1) Employ comprehensive neuropsychological assessments to detect early social cognitive and neuropsychological deficits. (2) Delineate neuro-functional substrates by using seed-based functional connectivity. Regions of interest (ROIs) to be used as seeds will be chosen based on observed social cognitive and neuropsychological deficits examined in the first step. (3) Examine specificity of neuro-functional signatures by comparing them with those of other frequent MS symptoms (e.g., fatigue).

## Materials and Methods

### Participants and Procedures

Thirty patients with relapsing–remitting MS (RRMS) and 34 healthy controls (HC) completed all cognitive assessments. Patients were recruited from the outpatient clinic at the Institute of Neuroimmunology and Multiple Sclerosis (INIMS), University Medical Centre Hamburg-Eppendorf. All patients had neurologist-confirmed RRMS according to McDonald criteria 2010 revisions (23) and were currently in remission. Only patients with low to moderate physical disability (EDSS ≤ 4.0) were included. MS patients were excluded if they had any neurological disorders (other than MS) or a lifetime diagnosis of any psychiatric disorder according to their medical history. Healthy controls were free of any neurological or psychiatric disorders. The study was approved by the ethics committee of the Hamburg Chamber of Physician and conducted in conformity with the 1954 Declaration of Helsinki (PV4356). All participants provided written informed consent prior to participation.

In order to minimize the impact of potential confounds, we used a fully automated algorithm (package optmatch, version 0.9.3) as implemented in R (version 3.1.3; http://www.r-project.org) to select 30 HC (out of 34 existing data sets) whose sex, age, and years of education best-matched those of MS patients (24) (see Table 1). All behavioral analyses are therefore based on 30 MS patients and 30 HC. For imaging analyses, two participants had to be excluded from fMRI-based analyses, one MS patient due to excessive head movement during the resting-state sequence (maximum translation > 2.5 mm) and one control subject due to technical problems during fMRI recordings. This resulted in a final sample of 29 MS patients and 29 controls for all imaging analyses.

**Table 1:** Demographical and clinical data of MS patients and healthy controls (HC).

	**MS**	**HC**	***p* (two-tailed)**
Age (years)	40.20 ± 9.87	39.57 ± 8.36	0.633
Sex (F:M)	18:12	19:11	0.795
Education (years)	12.17 ± 1.42	12.00 ± 1.44	0.167
Time between NP and SC test session (weeks)	8.27 ± 4.78	7.07 ± 4.61	0.326
Disease duration (years)	8.23 ± 5.04	–	–
EDSS (median, range)	1.75, 0–4	–	–
DMT (none/IFN/GA/natalizumab/fingolimod/DMF)	17/4/1/2/5/1	–	–
PBV	74.03 ± 2.68	75.89 ± 1.94	**0.016**
HADS-D anxiety	4.33 ± 3.12	3.07 ± 2.85	0.106
HADS-D depression	2.43 ± 2.90	1.60 ± 2.31	0.223
Q-IDS total (depression)	4.07 ±3.83	2.67 ± 2.41	0.097
FSMC total	50.00 ± 21.84	27.57 ± 6.76	**<0.001**
FSMC cognitive	25.20 ± 11.50	13.83 ± 3.90	**<0.001**
FSMC motor	24.80 ± 10.92	13.73 ± 3.25	**<0.001**
9-HPT dominant hand (time to complete in s)	18.78 ±2.15	17.72 ± 2.15	0.063

PBV, (brain volume/TIV)*100), volume of the brain tissue normalized by total intracranial volume (TIV), as segmented using FreeSurfer; MS, multiple sclerosis; HC, healthy control; NP, neuropsychological; SC, social cognition; EDSS, Expanded Disability Status Scale; DMT, disease-modifying therapies; IFN, interferon, GA, glatiramer acetate, DMF, dimethyl fumarate; PBV, percent brain parenchymal volume; HADS-D, Hospital Anxiety and Depression Scale-German; Q-IDS, Quick Inventory of Depressive Symptomatology; FSMC, Fatigue Scale for Motor and Cognitive Functions; 9-HPT, 9-Hole-Peg-Test.

*Bold values are significant at the p < 0.05 level*.

### Behavioral Data Collection

#### Social Cognition

We assessed social cognition by employing an ecological test battery encompassing the most relevant aspects of social cognition, theory of mind (ToM), emotion recognition (ER), and empathy. In the Movie for the Assessment of Social Cognition (MASC) (25), which was used to assess ToM, participants are required to infer mental states (i.e., thoughts, emotions, intentions) of four individuals who are shown in a 15-min video clip spending an evening together. During those 15 min, the movie is stopped 45 times asking participants multiple-choice questions about the protagonists' thoughts, emotions, and intentions.

We chose the FacePuzzle tasks to test implicit and explicit facial ER (26). The FacePuzzle tasks consist of 25 video clips each, showing professional actors display different basic as well as complex emotional states. In the implicit task, face videos are horizontally split below the eyes into eye and mouth videos, with eye videos being presented at the top of the screen and a selection of four mouth videos by the same actor at the bottom. Participants are asked to select the correct mouth video out of the four by moving it up to the eye clip at the top of the screen. Eye videos are played repeatedly from the start of the trial onward, whereas mouth videos only start playing once the computer mouse touches them. In this task, participants are not required to explicitly label the displayed emotions at any point. In the explicit task, in contrast, participants are to choose the correct out of four labels by dragging it onto a field below the target clip. Here, whole face videos are shown and again repeatedly played from the beginning of each trial until its completion. Participants were instructed to complete both tasks as fast and accurately as possible, but there was no time limit for any trials. As in the original publication (26), we used a composite measure consisting of response time for correct items divided by the number of correctly answered trials (“accuracy-adjusted response time”) as the main outcome measure in order to account for absence of a time limit.

Cognitive and emotional empathy was assessed by the Multifaceted Empathy Test (MET) (27). The MET consists of a series of photographs depicting people in emotionally charged situations such as a picture of a crying child. In the cognitive empathy part, participants are required to name the emotion that the person is experiencing in the picture by selecting one out of four given choices and is given immediate feedback on their selection. In the emotional empathy part of the MET, participants rate their emotional response to those pictures on a 7-point Likert scale.

#### Neuropsychological Assessment

A neuropsychological test battery was used to assess cognitive performance in MS patients and HC. For information processing speed, we used the oral version of the Symbol Digit Modalities Test (SDMT) (28). The Test Battery of Attentional Performance (TAP) (29) was used to measure attention. The WAIS-III Digit Span subtest was applied to measure different factors of working memory (30). The German version of the Auditory Verbal Learning Test (VLMT trials 1–5 and VLMT delayed recall) was administered to test verbal learning and memory (31) and the Brief Visuospatial Memory Test—Revised (BVMT) (32) to assess visuospatial learning and memory.

#### Other Relevant Behavioral Variables and Potential Confounds

In order to take other relevant behavioral variables and potential confounds into account, we also administered the Hospital Anxiety and Depression Scale—German version (HADS-D) (33) as well as the Quick Inventory of Depressive Symptomatology (Q-IDS) (34) to survey depression and the Fatigue Scale for Motor and Cognitive Functions (FSMC) (35) to measure fatigue-related symptoms. The 9-Hole-Peg-Test (9HPT) (36) of the dominant hand [as determined by the Edinburgh Handedness Inventory (37)] served as a measure of motor and sensory functioning.

### Functional Magnetic Resonance Imaging (fMRI) Data Collection

FMRI data were collected with a 3 Tesla Magnetom Trio scanner system (Siemens Medical Systems, Erlangen, Germany) using a 12-channel radiofrequency head coil. BOLD sequence: 40 transversal slices, image matrix = 94 × 94, *TR* = 2,500 ms, *TE* = 25 ms, flip angle = 90°, FOV = 175 × 175, voxel size = 2.65 × 2.65 × 2.65 mm^3^, slice thickness = 3 mm), which was composed of 250 volume acquisitions. Participants were instructed to lie still with their eyes focused on a fixation cross displayed at the center of the screen. MP-RAGE sequence: Image matrix = 232 × 288, repetition time (TR) = 2,500 ms, echo time (TE) = 2.12 ms, flip angle = 9°, field of view (FOV) = 192 × 239, slice thickness = 0.94 mm.

We also acquired a diffusion tensor imaging sequence (DTI) to examine structural tract integrity, and we used the MP-RAGE sequence to quantify regional brain volumes (atrophy) in MS and HC. Acquisition details for DTI and description of analysis are reported in the Supplementary Material.

### Data Analysis

#### Behavioral Data Analysis

As outlined above, MS patients and HC were matched for age, sex, and level of education to control effects of these variables. As there are no established cutoffs to define social cognition impairment in a diagnostic sense and normative data in many cases does not exist, we used raw performance scores in these tests as a continuous variable at the group level. Considering the approximate normal distribution of the variables and the sample size, we compared MS patients to controls by means of two-sample *t*-tests. After checking for outliers in the data, we explored relationships between decreased social cognitive dimensions and neuropsychological dimensions by means of Pearson correlation. IBM SPSS Statistics 22 for Windows (SPSS Inc., Chicago, IL, USA) was used for behavioral data analysis.

### Seed-Based Functional Connectivity (Resting-State fMRI)

A priori regions of interest (ROI) selection: Decreased performance was found in verbal learning (VLMT trials 1–5) and memory (VLMT delayed recall) as well as implicit emotion recognition (see behavioral results for details). Based on previous studies, we selected the hippocampus as a potential substrate for verbal learning and memory, (36) as well as the amygdala (AMY) and fusiform gyrus (FFG) due to their integral roles in emotion processing and facial emotion recognition, e.g., Adolphs (38). Bilateral masks of all regions of interest (hippocampus, FFG, AMY) were generated using the probabilistic Harvard–Oxford cortical and subcortical structural atlases in FSL (FMRIB; Software Library, www.fmrib.ox.ac.uk) (39) with a threshold of 50% and used as seed regions. For the FFG, the temporal occipital fusiform cortex mask was chosen.

Preprocessing and first level: Data preprocessing and single-subject analysis were performed using the advanced module of the data processing assistant for resting-state fMRI V3.2 (DPARSFA) (40), implemented in MATLAB R2014a and Statistical Parametric Mapping program 12 (SPM12; Wellcome Trust Center for Neuroimaging, London, UK). See Supplementary Materials for details on all applied preprocessing steps. Voxel-wise seed-based functional connectivity analysis was performed in MNI standard space using DPARSFA by computing the temporal correlation between the mean time series of both seed regions with the time series of every voxel in the brain. The correlation coefficients of each voxel were normalized to z-scores with Fisher's *r*-to-*z* transformation.

Second level: Employing a hypothesis-driven approach, we assumed that relevant functional connectivity networks would (a) show significant differences between MS and HC, (b) correlate with performance in behavioral tests, and (c) be specific to only one symptom domain. Therefore, FC group-level analyses followed a two-step procedure: First, two sample *t*-tests were performed to test for group differences of FC of the three selected seed regions. Seed regions that yielded significant FC alterations in MS were selected for further analysis. Second, we examined whether these regions that showed group differences in FC overlapped with regions related to implicit ER or verbal learning (VLMT trials 1–5) and memory (VLMT delayed recall). Therefore, voxel-wise multiple-regression models were computed for these regions. Analyses were carried out using FSL FLAMEO separately for each seed region. Multiple-comparison corrections at the cluster level were performed on the whole brain based on Gaussian random field theory (minimum *z* > 2.3; corrected cluster significance *p* < 0.05).

### DTI and Volumetry

See Supplementary Material 1.2 for details on DTI data and volumetry. In short, we analyzed the diffusion-weighted images using the Diffusion Toolbox implemented in FSL 5.0.9. A diffusion tensor model was fitted to each voxel with the appropriate steps. Fractional anisotropy (FA) and mean diffusivity (MD) maps were calculated for each participant. Statistical between-group analyses of diffusion-weighted data were performed using tract-based spatial statistics (TBSS) (41) and the randomized tool (42) with 5,000 permutations. Automated volumetry was performed using the FreeSurfer image analysis suite 6.0 (http://surfer.nmr.mgh.harvard.edu/). Whole-brain gray matter volume and hippocampus, amygdala, and FFG volumes were estimated.

## Results

### Social Cognition and Neuropsychological Performance

For details on participants' clinical and demographical data, see Table 1. As expected given the low to moderate disease severity, the patient cohort was characterized by only subtle decreases in cognitive performance. Significantly decreased performance in MS compared to HC was observed only in two domains: Verbal learning and memory (VLMT trials 1–5, *t*_50.462_ = 2.229, *p* = 0.030; VLMT delayed recall *t*_49.070_ = 2.246, *p* = 0.029), and implicit emotion (ER) recognition (FacePuzzle *t*_48.484_ = 2.634, *p* = 0.011); see Table 2. Largest effect sizes were observed for implicit ER (d_cohen_ = 0.680), closely resembling effect sizes for such deficits reported for MS in recent meta-analyses (10, 11).

**Table 2:** Social cognition and neuropsychological performance data of MS patients and healthy controls (HC).

	**MS**	**HC**	***P***	**d_**Cohen**_**
MASC (sum correct)	11.47 ± 2.15	12.27 ± 1.95	0.242	0.390
MET				
Cognitive empathy (sum correct)	31.20 ± 3.99	32.80 ± 3.21	0.093	0.442
Emotional empathy (sum)	5.63 ± 1.32	5.94 ± 1.33	0.388	0.234
FP (accuracy adjusted RT in s)^*^				
Implicit	0.80 ± 0.29	0.64 ± 0.18	**0.011**	**0.680**
Explicit	0.18 ± 0.07	0.15 ± 0.05	0.064	0.489
SDMT (sum correct)	61.23 ± 15.15	64.97 ± 14.61	0.335	0.251
VLMT (sum)				
Correctly remembered words (trials 1–5)	58.93 ± 9.27	63.47 ± 6.17	**0.030**	**0.577**
Forgotten words (trials 5–7)	1.23 ± 2.13	0.20 ± 1.35	**0.029**	**0.578**
BVMT-R (sum)
Total learning	22.66 ± 6.99	24.53 ± 5.70	0.262	0.293
Delayed recall	8.76 ± 2.28	9.03 ± 1.59	0.592	0.138
Recognition: yes/no hits	5.69±.541	5.83±.539	0.335	0.259
Recognition: yes/no false positive	0.03 ± 0.186	0.00 ± 0.00	0.322	0.228
Digit symbol (sum correct)				
Forward	8.93 ± 1.74	9.73 ± 2.02	0.105	0.425
Backward	8.70 ± 2.05	8.67 ± 1.73	0.946	0.016
TAP (RT in ms)^*^				
Alertness—without warning tone	262.53 ± 44.13	242.63 ± 31.65	0.050	0.518
Alertness—with warning tone	251.07 ± 38.61	240.20 ± 31.54	0.237	0.308
Covert shift of attention—valid	291.80 ± 54.04	374.50 ± 44.73	0.182	0.349
Covert shift of attention—invalid	335.45 ± 58.89	308.93 ± 50.80	0.069	0.482
Incompatibility–compatible condition	454.83 ± 77.11	426.33 ± 68.63	0.136	0.390
Incompatibility–incompatible condition	500.47 ± 94.10	469.90 ± 68.48	0.160	0.371

* *Lower score indicates better performance*.

*MASC, Movie for the Assessment of Social Cognition; MET, Multifaceted Empathy Test; FP, FacePuzzle task; SDMT, Symbol Digit Modalities Test; VLMT, Auditory Verbal Learning Test (Verbaler Lern-und Merkfähigkeitstest); BVMT-R, Brief Visuospatial Memory Test-Revised; TAP, Test battery of Attentional Performance; MS, multiple sclerosis; HC, healthy control*.

*Bold values are significant at the p < 0.05 level*.

We observed no significant associations between decreased performance in social cognition and decreased performance in verbal learning and memory (see Supplementary Table 1).

### Functional Connectivity Signatures

**Figure 1:**
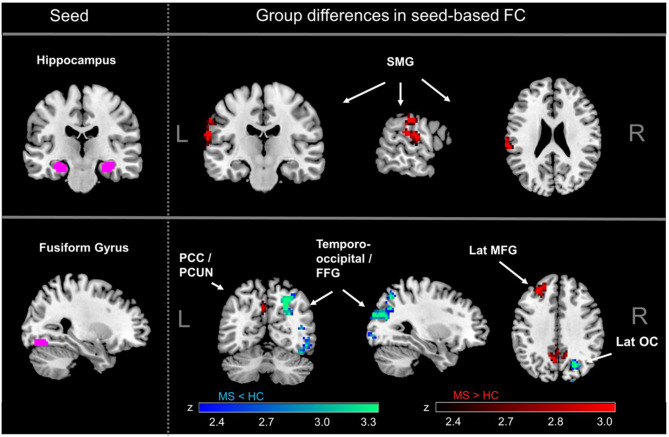
MS-related differences in seed-based functional connectivity (FC). Significantly increased connectivity in MS compared to healthy controls was observed for the hippocampus, mainly with the supramarginal gyrus (SMG). The fusiform gyrus (FFG) showed lower connectivity in MS compared to controls in occipital regions and increased connectivity with PCC and PCUN. There were no significant differences for MS vs. healthy controls for FC of the amygdala. SMG, supramarginal gyrus; PCC, posterior cingulate cortex; PCUN, precuneus; FFG, fusiform gyrus; lat MFG, lateral middle frontal gyrus; lat OC, lateral occipital gyrus.

We observed significant group differences in seed-based functional connectivity (sbFC) of the fusiform gyrus (FFG) as well as the hippocampus in MS patients compared to controls (Figure 1), whereas there were no significant differences detected in functional connectivity of the amygdala (AMY). In line with previous studies in early MS (43), connectivity between hippocampus and the left supramarginal gyrus was significantly higher in MS compared to controls (adjusted for multiple comparisons). Moreover, the FFG connectivity to the bilateral posterior cingulate cortex reaching into the precuneus and the left lateral middle frontal gyrus was also significantly higher in MS. FFG connectivity to the occipital part of the right FFG and to the right lateral occipital cortex (OC) was significantly lower in MS.

**Figure 2:**
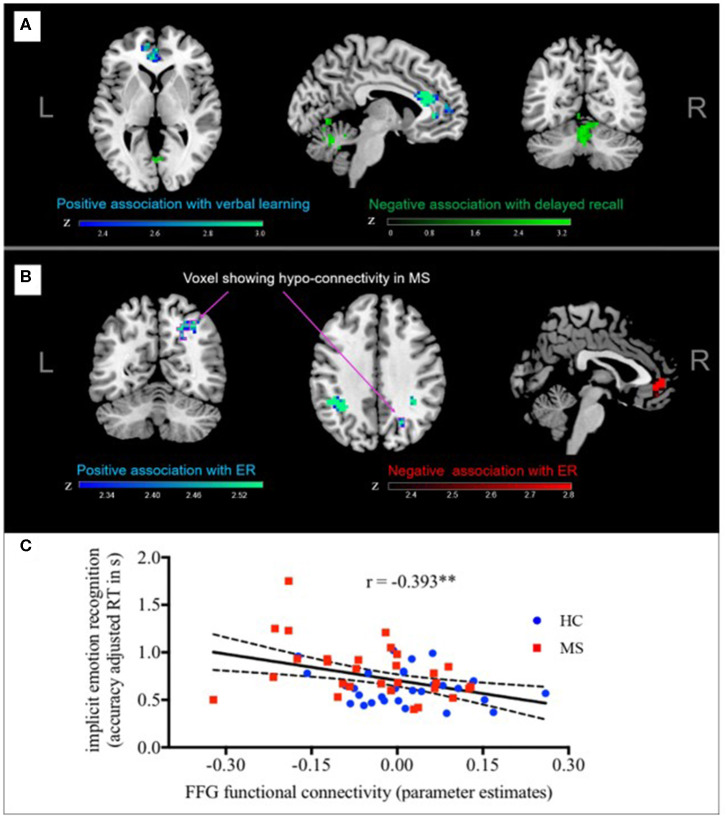
Functional connectivity and performance in implicit ER. Fusiform gyrus-FC with MPFC was negatively associated with implicit ER, whereas fusiform gyrus-FC with left angular gyrus, and right lateral OC was positively associated with implicit ER. Arrows point to the cluster of voxels in the right lateral OC that meets our criteria for a distinct neural signature of implicit ER deficits in MS since **(A)** functional connectivity to the FFG was significantly reduced in MS patients compared to controls and **(B)** lower FC to the FFG was associated with poorer implicit ER performance. **(C)** For illustrative purposes, a scatterplot depicting the correlation between individual implicit ER (accuracy adjusted RT) and functional connectivity of the right later OC with the fusiform gyrus is shown. FC, functional connectivity; ER, emotion recognition; FFG, fusiform gyrus. **p < 0.01.

Second, we explored if functional connectivity networks found to be different between MS and HC were also related to performance in learning/memory or implicit ER. In the case of the hippocampus, there was no overlap between regions where FC was related to verbal learning (VLMT trials 1–5) and verbal memory (VLMT delayed recall) and those regions where significant group differences were detected. However, results showed that for fusiform gyrus connectivity, there was a cluster of voxels in the right lateral OC that showed (a) significantly decreased connectivity to the fusiform gyrus in MS patients compared to controls and (b) where lower functional connectivity to the fusiform gyrus was associated with poorer implicit ER performance (Figure 2 and Supplementary Figure 1). Thus, functional connectivity between fusiform gyrus and right lateral OC meets our criteria for a neural signature of implicit ER deficits in MS. Besides these voxels, fusiform gyrus FC with MPFC was negatively associated with implicit ER, whereas fusiform gyrus FC with left angular gyrus and right lateral OC was positively associated with implicit ER.

### Potential Confounds Related to MS-Related Symptom Domains

Finally, we aimed to examine if functional connectivity signatures described above are specific to social cognition (i.e., implicit ER) when compared to other frequent MS symptoms that might impact performance on these tests, such as motor and sensory functioning, depression, anxiety, or fatigue.

Specificity on behavioral level: As expected, our patient cohort showed higher levels of fatigue (FSMC) and a trend toward impaired motor and sensory function of the dominant hand (9HPT); see Table 1. Since psychiatric comorbidities were exclusion criteria for this study, we did not observe group differences in anxiety or depressive symptoms. Small to moderate negative linear relationships were found between fatigue and ER performance (e.g., FSMC total and accuracy adj. RT in implicit FP: *r* = 0.348, *p* = 0.006), which tended to be driven by cognitive rather than motor-related fatigue symptoms. There was also a small correlation between motor fatigue and delayed recall in the VLMT, but not to verbal learning. Moreover, motor and sensory function (9HPT) correlated positively with both ER (implicit and explicit) and verbal learning. Detailed results of correlational analyses between fatigue and motor function, on the one hand, and ER and verbal memory, on the other, is found in Supplementary Table 1.

Specificity on neural level: As a next step, we compared neural mappings of fatigue and motor function to those of implicit ER and verbal memory. We again computed multiple regression models to assess correlations between sbFC and (a) total fatigue (sum FSMC) as well as motor/sensory function (9HPT). Fatigue scores correlated positively with FFG-based FC to the MPFC and negatively with FFG-based FC to right lateral PFC. Fatigue scores were not significantly correlated with hippocampus-based FC across the brain. Motor and sensory function (9HPT) was negatively correlated with FFG-based FC to the lateral temporal gyrus, positively with hippocampus-based FC to the cerebellum, and negatively with hippocampus-based FC to the precentral gyrus (extending into the supplementary motor cortex). Intriguingly, as shown in Supplementary Figure 1, functional connectivity maps for fatigue, motor/sensory function, and implicit ER showed very little overlap, which was restricted to the medial prefrontal cortex. Notably, the described right lateral OC that was related to social cognitive deficits did not show any association with these other measures.

### Structural Measures

MS patients showed subtle but widespread white matter abnormalities (i.e., fractional anisotropy, FA). In line with published data (20), lower performance in implicit ER (FacePuzzle) was correlated with lower values of FA across widespread white matter networks of both hemispheres. See Table 1 for percent brain parenchymal volume (PBV). Moreover, whole-brain volume was significantly smaller in MS patients (i.e., showed significant atrophy); see Supplementary Material 1.2.1 and Supplementary Figure 2. Whole-brain volume was not correlated with any of the cognitive MS symptom-related measures. There were no significant group differences in any of the regions of interest (see Supplementary Material 1.2.2 and Supplementary Table 2).

## Discussion

In this study, we identified distinct pathophysiological underpinnings of social cognition deficits (implicit emotion recognition) by means of seed-based functional connectivity analysis in multiple sclerosis. On the behavioral level, we observed decreased performance in implicit ER that was independent of decreased performance in other cognitive areas such as verbal learning and memory. Results from previous studies investigating associations between standard neurocognitive domains (e.g., executive functions) and social cognition on the behavioral level have been inconsistent. Whereas some report medium-sized correlations between social cognition and executive functions (44, 45), others argue in favor of dissociable symptom clusters that arise independently from each other (46). Our sample was characterized by low to medium disease severity; it is likely that deficits in cognitive functions are largely intercorrelated when the disease progresses to a later stage. In line with this idea, implicit general and social cognition abilities have been suggested to be a developmental precursor to explicit (social) cognitive abilities (26, 47–49). Previous studies suggest a behavioral dissociation between implicit and explicit aspects of social cognition, with impairments highlighted on implicit dimensions in CNS disorders with socio-affective impairments such as autism spectrum disorder [e.g., (26, 50, 51)]. Moreover, implicit measurements of emotion recognition do not require individuals to choose a proper term for a given facial affect picture. These kinds of implicit measurements might thus be more suitable to disentangle social cognition deficits from deficits in other cognitive domains such as verbal learning and memory. Differences between tests performed in previous investigations, particularly in social cognition tests, might therefore have additionally influenced heterogeneous results concerning the relationship between different cognitive symptom clusters in MS.

Functional connectivity analyses revealed distinct correlates. In particular, fusiform gyrus (FFG) FC to the right lateral occipital cortex was both positively related to implicit ER performance and significantly reduced in MS patients compared to controls. Contrasting this resting-state-based functional connectivity signature to those functional patterns related to other common MS symptoms (i.e., fatigue, motor/sensory function deficits, depression, anxiety) showed no overlap, lending further support to its function as distinct neural substrate. The correlation of reduced implicit ER with levels of fatigue has previously been interpreted as an indication for possible common neural networks that might underlie these symptoms (52). In general, functional connectivity maps for fatigue, motor/sensory function, and implicit ER showed very little overlap, restricted to the medial prefrontal cortex (MPFC). Fatigue and motor function impairments have been shown to be related to sensorimotor networks (53, 54). In the case of fatigue, connectivity changes in fundamental motivational and reward networks are believed to be additionally implicated in its pathophysiology (55). The connectivity between fusiform gyrus and MPFC is essential for the evaluation of affective states at higher and lower levels, which likely figures prominently in both complex implicit emotion recognition and fatigue in MS. Here, we demonstrate that functional connectivity analysis represents a promising approach to dissect neural signatures of symptoms that are intercorrelated on a behavioral level.

The distinct substrates of implicit emotion recognition deficits in MS as identified in our study, namely, a functional network involving the FFG and the lateral occipital cortex, are in line with the known role of these regions in emotion and social cognition. The FFG and occipital cortex are both integral parts of the cortical face processing network (56). The occipital cortex is likely to support attentional control of perceptual inputs in an early stage of emotion processing (57), while the FFG has been implicated in more general emotional reactivity (58). Both have previously been found to show altered functional activity patterns in patient groups characterized by altered emotional control. For example, reduced activation of the right occipital cortex during emotional picture processing was found to be a potential initiating factor for cognitive disorder in depressed patients (59). In another study, the FFG was found to be hypo-activated in patients with borderline personality disorder (BPD) during processing of negative pictures.

Only few studies to date have explored the functional connectivity substrates of social cognition deficits in MS. In line with preceding structural imaging studies, complementary analyses replicated the non-specific associations of social cognition deficits with widespread structural CNS damage in MS (20). In contrast to previous studies, we were able to contrast social cognition with other cognitive symptoms and compare functional correlates in the brain. Thus, our study provides novel insight into the pathophysiology of these deficits, indicating that early manifestations of deficits in a higher level, that is, highly complex cognitive functions such as social cognition, are specifically linked to functional network alterations.

Several limitations should be noted. First, although our analysis suggests that deficits in social cognition (i.e., emotion recognition) in MS are not simply an epiphenomenon of more basic cognitive dysfunction and may arise independently, the trajectories of the evolution of these symptoms on a behavioral as well as CNS level need to be established in longitudinal studies. Moreover, as there are no established cutoffs to define social cognition impairment in a diagnostic sense, we used performance in these tests as a continuous variable at the group level. Furthermore, due to the high variety of basic and complex, naturalistic video-based material (60), it was not possible to reliably analyze the impact of distinct emotions (e.g., fear vs. happy) on behavioral and neural read-outs. Lastly, while we used appropriate thresholding to ensure validity of our results, sample size is relatively small and we used a comparatively liberal threshold for multiple comparisons in this exploratory study. Thus, future studies should aim to replicate and expand the results.

To sum up, social cognitive deficits in MS seem to be related to distinct neural connectivity patterns. Establishing these kinds of subtle relationships between neural signatures and behavioral categories is vital for advancing knowledge about their underlying pathobiology (7). The approach of finding distinct neural signatures of fine-grained behavioral phenomena characteristic of certain CNS disorders has already been fruitful in several other research areas including addiction, panic disorder, and autism (61–64). However, due to the relatively small sample size, replication in larger studies will be needed to confirm this result. If replication is successful, future studies might investigate whether the neural substrates are specific to MS. The symptom domains we have explored here in MS are frequently seen in many other CNS disorders across diagnostic boundaries. This will require studying patient groups from different diagnostic categories but largely overlapping symptoms (e.g., social cognition deficits and/or deficits in learning and memory). Finally, it will be important to explore if our findings have therapeutic implications, particularly with regard to rehabilitation.

## Data Availability Statement

The raw data supporting the conclusions of this article will be made available by the authors upon request, without undue reservation.

## Ethics Statement

The studies involving human participants were reviewed and approved by Ethics committee of the Hamburg Chamber of Physicians, Hamburg, Germany. The patients/participants provided their written informed consent to participate in this study.

## Author Contributions

SMG, AE, CH, IK-P, ID, and CF: study design and acquisition of funding. JP and SL: data acquisition. SG, JH, MM, and J-PS: data processing and analysis. SG and JH: statistical analysis. SG and SMG: writing of manuscript. JH, JP, MM, SL, KW, CO, IK-P, AE, CH, J-PS, ID, and CF: revising manuscript. All authors contributed to the article and approved the submitted version.

## Conflict of Interest

The authors declare that the research was conducted in the absence of any commercial or financial relationships that could be construed as a potential conflict of interest.
