# Supplementary material for: Distinct Functional Connectivity Signatures of Impaired Social Cognition in Multiple Sclerosis
Source: Front Neurol. 2020 Jun 25;11:507. doi: 10.3389/fneur.2020.00507 (PMC7330009; doi:10.3389/fneur.2020.00507)
Supplement: Supplementary file 1 [file Data_Sheet_1.docx]

Supplementary Material

# Supplementary Materials and Methods

## Preprocessing of functional MRI data

Data preprocessing was performed using the advanced module of the data processing assistant for resting state fMRI V3.2(DPARSFA),[1] implemented in MATLAB R2014a and Statistical Parametric Mapping program 12 (SPM12; Wellcome Trust Center for Neuroimaging, London, UK). The first four volumes were discarded to ensure magnetization equilibrium, the remaining functional volumes were corrected for differences in slice acquisition timing and individual time series were realigned. Cropping and brain extraction was performed on T1-weighted MPRAGE images, before they were co-registered to the mean functional image. Transformed structural images were then segmented into GM, WM and CSF and the Friston 24-parameter model was used for removing nuisance signals by means of regressing out parameters of head motion from the realignment step. The signals from WM and CSF were regressed out to reduce respiratory and cardiac effects. The DARTEL algorithm[2] was used to normalize / register individual data to MNI standard space and then data were smoothed with a 4 mm full-width-half-maximum (FWHM) Gaussian kernel. Further, nuisance regression was performed where time points with framewise displacement (FD) > 0.5 mm[3] as well as one previous and two subsequent volumes were included as regressors. Global signal regression was not performed. Finally, the time series was temporally filtered (0.01–0.1 Hz). The quality control module of dpabi (Data Processing & Analysis of Brain Imaging toolbox)[1] was used to visually inspect all functional and structural images as well as their normalization. A threshold of maximum head motion > 2.5 mm resulted in the exclusion of one MS patient, neither mean absolute motion nor mean FD (Jenkinson) differed between the groups (absolute motion: p = .65; FD: p = .45).

## Structural MRI and Analysis

### **Diffusion Tensor Imaging (DTI)**

Acquisition parameters: A single-shot echo planar imaging DTI sequence was applied (repetition time (TR)/echo time (TE) = 7200/87 ms; field of view (FOV) = 240 mm; matrix 96 × 96, 50 slices without gap, slice thickness = 2 mm, 64 non-colinear directions, b-value = 1000 s/mm2, parallel imaging generalized auto-calibrating partially parallel acquisitions (GRAPPA) factor 2).

Processing and analysis: We analyzed the diffusion weighted images using the Diffusion Toolbox implemented in FSL 5.0.9 (FMRIB’s Software Library; http://www.fmrib.ox.ac.uk/fsl). Diffusion images were brain extracted and corrected for eddy current distortions and motion artefacts. Each preprocessed image was visually inspected to ensure data quality. Next, a diffusion tensor model was fitted to each voxel, resulting in fractional anisotropy (FA) and mean diffusivity (MD) maps for each participant. Each individual map was aligned to the representative study-specific target image, non-linearly registered to MNI152 standard space (1x1x1mm), and averaged to create the FA and MD skeletons on which the individual data are projected.

Statistical between-group analyses of diffusion-weighted data was performed using tract-based spatial statistics (TBSS)[4] and the randomize tool[5] with 5000 permutations as implemented in FSL. Threshold-free cluster enhancement was used to correct for multiple comparisons. Correlational analyses were similarly modelled within the FSL general linear model toolbox.

Results: MS patients showed subtle, but widespread white matter abnormalities as measured by decreases in fractional anisotropy (FA; supplementary Figure 2A). Lower performance (i.e. longer reaction times) in implicit ER (FacePuzzle) was unspecifically correlated with lower values of FA across widespread white matter networks of both hemispheres (supplementary Figure 2B).

### Cortical and subcortical volumetry

Processing and analysis: The T1-weighted images (MP-RAGE) were preprocessed, including motion correction, removal of non-brain tissue, and intensity normalization. The individual scans were subsequently transformed to Talairach coordinates. The individual volumes were scaled for intracranial volume (ICV) in order to correct for overall head size effects using the following formula: Volumeadjusted = Volumeobserved – β (ICVobserved – ICVsample mean); with β being defined as the slope of the linear regression of ICV on the respective ROI volume^[6; 7]^Please see Table 1 for Percent brain parenchymal volume (PBV), which represents the volume of the brain tissue normalized by total intracranial volume (TIV), as segmented using Freesurfer (PBV = (brain volume/TIV)*100). Automated volumetry of the ROIs was performed using the Freesurfer image analysis suite 6.0 (http://surfer.nmr.mgh.harvard.edu/). As a first step, whole brain grey matter volume was estimated. Subsequently, volumes of the deep grey matter structures, i.e. the hippocampus and amygdala, were derived from subcortical segmentation and FFG volume was estimated using the cortical parcellation as implemented in Freesurfer’s surface-based stream. The automated segmentations were visually controlled to ensure their quality and volumes were scaled to intracranial volume (ICV) to correct for head size. For each ROI volume, results for both hemispheres were added to give bilateral grey matter volume sizes.

Two sample *t*-tests were performed to test for group differences. Relationships between volumetric data and neuropsychological tests, measures of social cognition and other MS-related symptoms were explored by means of Pearson correlations.

Results: Whole brain volume was significantly smaller in MS patients, that is, brains of the MS group showed significant atrophy. However, it did not correlate with neuropsychological tests, measures of social cognition or other MS related symptoms. Despite the fact that all examined regional brain volumes were descriptively smaller in MS patients, there were no significant group differences in any of the regions (see supplementary Table 2).

# Supplementary Figures and Tables

## Supplementary Figures


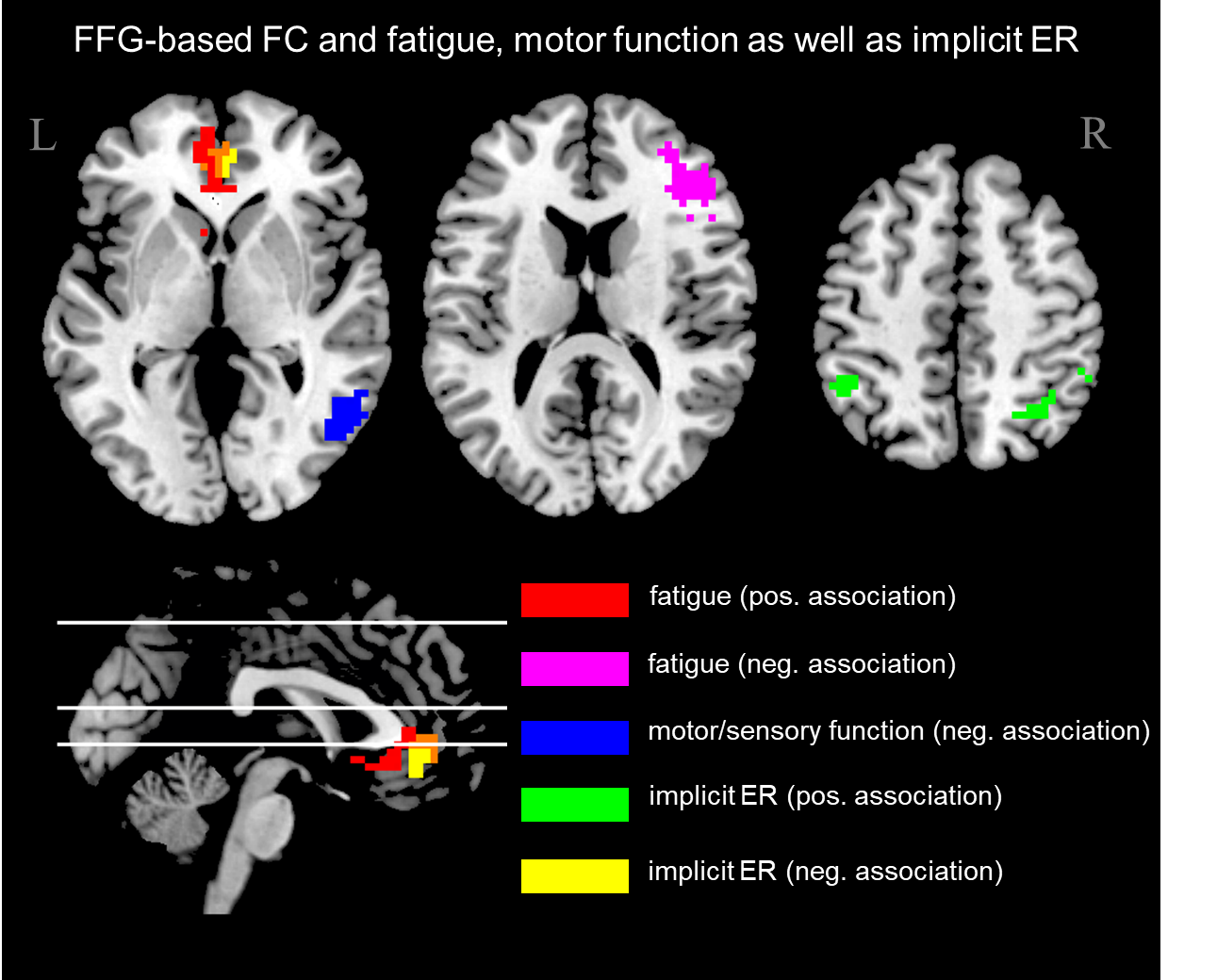


**Supplementary Figure 1.** **Functional connectivity signatures of MS-related symptoms in motor and sensory function, fatigue and social cognition deficits.** Comparison between brain areas showing fusiform gyrus-based FC association with fatigue and those that show an association with implicit ER. Resulting functional connectivity maps for fatigue, motor/sensory function, and implicit ER showed few overlapping voxels restricted to the medial prefrontal cortex only. *Abbreviations:* FFG = fusiform gyrus, FC = functional connectivity, ER = emotion recognition


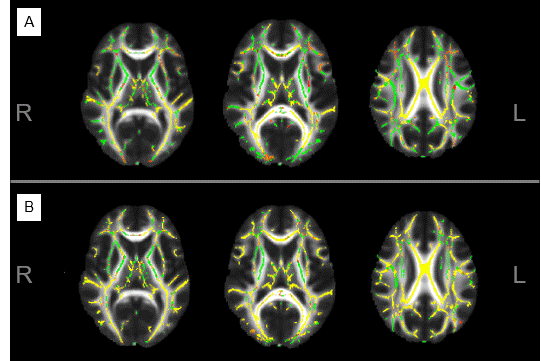


**Supplementary Figure 2.** A. White matter tracts with significant group differences (red-yellow) in fractional anisotropy (FA) between Multiple Sclerosis (MS) patients and controls are shown overlaid on the mean FA skeleton (green). MS patients showed subtle, but widespread FA reductions. B. White matter tracts with significant negative correlation (red-yellow) between FA and implicit emotion recognition (accuracy adjusted reaction time) are shown overlaid the mean FA skeleton (green). In line with recently published data,[8] lower performance (i.e. longer reaction times) in implicit emotion recognition (FacePuzzle) was correlated with lower values of FA across widespread white matter networks of both hemispheres.

## Supplementary Tables

***Table 1.*** Correlation coefficients between neuropsychological tests, measures of social cognition and other MS-related symptoms (motor and sensory function, depression, fatigue).

|  | **1** | **2** | **3** | **4** | **5** | **6** | **7** | **8** | **9** | **10** | **11** |
| --- | --- | --- | --- | --- | --- | --- | --- | --- | --- | --- | --- |
| **1 FP explicit** (aaRT in s) | --- |  |  |  |  |  |  |  |  |  |  |
| **2 FP implicit** (aaRT in s) | **.592**** | --- |  |  |  |  |  |  |  |  |  |
| **3 VLMT** **correctly remembered words** (sum trials1-5) | -.101 | .097 | --- |  |  |  |  |  |  |  |  |
| **4 VLMT** **forgotten words in delayed recall** (trials 5-7) | .106 | .225 | **-.421**** | --- |  |  |  |  |  |  |  |
| **5 TAP alertness – without warning tone** (RT in s) | .027 | .167 | .002 | .069 | --- |  |  |  |  |  |  |
| **6 HADS-D Anxiety** | .235 | **.424**** | -.006 | .021 | **.377**** | --- |  |  |  |  |  |
| **7 HADS-D Depression** | **.255*** | .213 | -.166 | **.256*** | .039 | **.483**** | --- |  |  |  |  |
| **8 Q-IDS total (Depression)** | **.338**** | **.427**** | -.050 | .128 | .223 | **.697**** | **.641**** | --- |  |  |  |
| **9 FSMC total** | **.277*** | **.348**** | -.117 | .219 | **.383**** | **.587**** | **.545**** | **.661**** | --- |  |  |
| **10 FSMC cognitive** | **.339**** | **.388**** | -.107 | .171 | **.386**** | **.613**** | **.554**** | **.707**** | **.982**** | --- |  |
| **11 FSMC motor** | .200 | **.292*** | -.123 | **.261*** | **.365**** | **.537**** | **.515**** | **.587**** | **.980**** | **.923**** | --- |
| **12 9-HPT dominant hand** (time to complete in s) | **.280*** | **.311*** | **-.396**** | **.307*** | .125 | **.366**** | .222 | **.497**** | **.387**** | **.418**** | **.338**** |

*Note:* Pearson correlation coefficients (*r*), ** = *p* <.01, * = *p* <.05; *Abbreviations:* FP = Face Puzzle task; aaRT = accuracy adjusted reaction time; VLMT = Auditory Verbal Learning Test (*Verbaler Lern-und Merkfähigkeitstest*); TAP = Test battery of Attentional Performance, HADS-D = Hospital Anxiety and Depression Scale-German; Q-IDS = Quick Inventory of Depressive Symptomatology; FSMC = Fatigue Scale for Motor and Cognitive Functions; 9-HPT = 9-Hole-Peg-Test

***Table 2.*** Results of additional automated volumetry (see supplementary methods 1.2.2) for the whole brain and all regions of interest (i.e. amygdala, hippocampus, fusiform gyrus) compared between MS patients and healthy controls.

| **ICV** | **MS** | **HC** | ***p*** (two-tailed) | **d_Cohen_** |
| --- | --- | --- | --- | --- |
| **Whole brain** | 1107790.52 ± 135207.29 | 1198987.70 ± 134497.23 | .013 | .676 |
| **Amygdala** | 3121.05 ± 419.74 | 3217.98 ± 390.21 | .366 | .239 |
| **Hippocampus** | 7920.45 ± 855.50 | 8080.93 ± 954.82 | .503 | .177 |
| **Fusiform gyrus** | 19295.30 ± 2486.41 | 20349.76 ± 2579.98 | .119 | .416 |

*Abbreviations:* MS = Multiple Sclerosis; HC = healthy control; ICV = intracranial volume

# References

[1] C.-G. Yan, X.-D. Wang, X.-N. Zuo, and Y.-F. Zang, DPABI: Data Processing & Analysis for (Resting-State) Brain Imaging. Neuroinformatics 14 (2016) 339-351.

[2] J. Ashburner, A fast diffeomorphic image registration algorithm. NeuroImage 38 (2007) 95-113.

[3] J.D. Power, K.A. Barnes, A.Z. Snyder, B.L. Schlaggar, and S.E. Petersen, Spurious but systematic correlations in functional connectivity MRI networks arise from subject motion. NeuroImage 59 (2012) 2142-2154.

[4] S.M. Smith, M. Jenkinson, H. Johansen-Berg, D. Rueckert, T.E. Nichols, C.E. Mackay, K.E. Watkins, O. Ciccarelli, M.Z. Cader, P.M. Matthews, and T.E.J. Behrens, Tract-based spatial statistics: Voxelwise analysis of multi-subject diffusion data. NeuroImage 31 (2006) 1487-1505.

[5] A.M. Winkler, G.R. Ridgway, M.A. Webster, S.M. Smith, and T.E. Nichols, Permutation inference for the general linear model. NeuroImage 92 (2014) 381-397.

[6] B. Fischl, A. van der Kouwe, C. Destrieux, E. Halgren, F. Ségonne, D.H. Salat, E. Busa, L.J. Seidman, J. Goldstein, D. Kennedy, V. Caviness, N. Makris, B. Rosen, and A.M. Dale, Automatically Parcellating the Human Cerebral Cortex. Cereb Cortex 14 (2004) 11-22.

[7] B. Fischl, D.H. Salat, E. Busa, M. Albert, M. Dieterich, C. Haselgrove, A. van der Kouwe, R. Killiany, D. Kennedy, S. Klaveness, A. Montillo, N. Makris, B. Rosen, and A.M. Dale, Whole Brain Segmentation. Neuron 33 (2002) 341-355.

[8] S. Batista, C. Alves, O.C. d’Almeida, A. Afonso, R. Félix-Morais, J. Pereira, C. Macário, L. Sousa, M. Castelo-Branco, I. Santana, and L. Cunha, Disconnection as a mechanism for social cognition impairment in multiple sclerosis. Neurology 89 (2017) 38-45.
